# Enteral liquid ventilation oxygenates a hypoxic pig model

**DOI:** 10.1016/j.isci.2023.106142

**Published:** 2023-02-13

**Authors:** Tasuku Fujii, Yosuke Yoneyama, Akiko Kinebuchi, Naoki Ozeki, Sho Maeda, Norikazu Saiki, Toyofumi Fengshi Chen-Yoshikawa, Hiroshi Date, Kimitoshi Nishiwaki, Takanori Takebe

**Affiliations:** 1 Department of Anesthesiology, Nagoya University Graduate School of Medicine, 65 Tsurumai-cho, Showa-ku, Nagoya 466-8550, Japan; 2 Institute of Research, Tokyo Medical and Dental University (TMDU), 1-5-45 Yushima Bunkyo-ku, Tokyo 113-8510, Japan; 3 Department of Thoracic Surgery, Nagoya University Graduate School of Medicine, 65 Tsurumai-cho, Showa-ku, Nagoya 466-8550, Japan; 4 Department of Thoracic Surgery, Graduate School of Medicine, Kyoto University, 54 Shogoin-Kawahara-cho, Sakyo-ku, Kyoto 606-8507, Japan; 5 Division of Gastroenterology, Hepatology & Nutrition, Developmental Biology, Center for Stem Cell and Organoid Medicine (CuSTOM). Cincinnati Children’s Hospital Medical Center, 3333 Burnet Avenue, Cincinnati, OH 45229-3039, USA; 6 Department of Pediatrics, University of Cincinnati College of Medicine, 3333 Burnet Avenue, Cincinnati, OH 45229-3026, USA; 7 Premium Research Institute for Human Metaverse Medicine (WPI-PRIMe), and Division of Stem Cell and Organoid Medicine, Osaka University, Suita, Osaka 565-0871, Japan

**Keywords:** Gastroenterology, Therapy, Porcine respiratory medicine

## Abstract

The potential of extrapulmonary ventilation pathways remains largely unexplored. Here, we assessed the enteral ventilation approach in hypoxic porcine models under controlled mechanical ventilation. 20 mL/kg of oxygenated perfluorodecalin (O_2_-PFD) was intra-anally delivered by a rectal tube. We simultaneously monitored arterial and pulmonary arterial blood gases every 2 min up to 30 min to determine the gut-mediated systemic and venous oxygenation kinetics. Intrarectal O_2_-PFD administration significantly increased the partial pressure of oxygen in arterial blood from 54.5 ± 6.4 to 61.1 ± 6.2 mmHg (mean ± SD) and reduced the partial pressure of carbon dioxide from 38.0 ± 5.6 to 34.4 ± 5.9 mmHg. Early oxygen transfer dynamics inversely correlate with baseline oxygenation status. SvO_2_ dynamic monitoring data indicated that oxygenation likely originated from the venous outflow of the broad segment of large intestine including the inferior mesenteric vein route. Enteral ventilation pathway offers an effective means for systemic oxygenation, thus warranting further clinical development.

## Highlights

• Enteral liquid ventilation oxygenates independent of native lung function
• Early oxygen transfer dynamics inversely correlate with baseline oxygenation status
• Enteral liquid ventilation reduced the partial pressure of carbon dioxide
• Oxygenation commences from the venous outflow of the large bowel

## Graphical abstract

**Figure:**
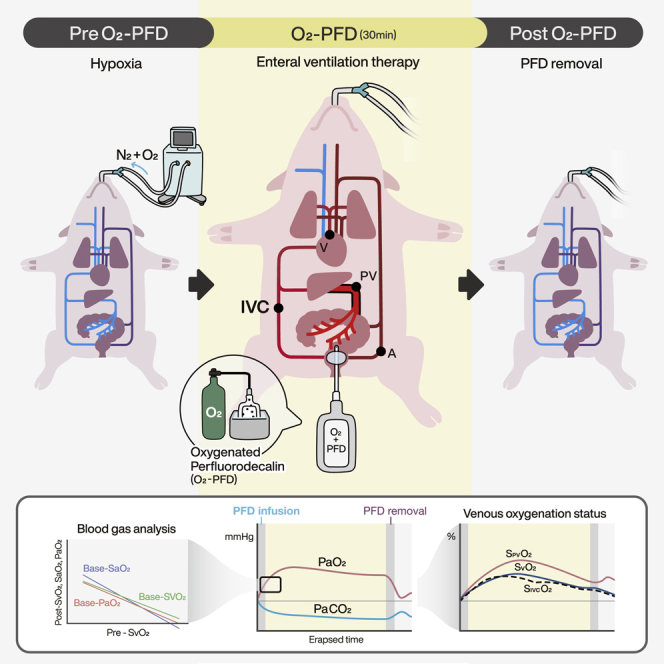

## Teaser

Gastroenterology; Therapy; Porcine model; Respiratory medicine; Enteral ventilation.

## Introduction

For progressive respiratory failure, oxygen therapy can save the patient’s life. However, approximately one-quarter of hospitals surveyed in resource-limited countries lack sufficient oxygen supply.^1^ This limitation is becoming more obvious with recent COVID-19 pandemic as patients regularly require interhospital transport for diagnostic or therapeutic purposes accelerated by hospital overcrowding. Although there are transient oxygen delivery methods available, several major limitations are important to take into account. For example, patients are at risk for severe respiratory distress or exhaustion due to increased breathing load.^2^ Therefore, a simple and less invasive lung-independent respiratory support is highly demanding particularly in areas with resource-poor settings.

Historically, extrapulmonary oxygenation routes involving the gastrointestinal (GI) tract have been clinically explored. In the 1950s, for example, gastric gas insufflation was tested to treat asphyxia in newborns.^3^^,^^4^^,^^5^^,^^6^^,^^7^ However, the effectiveness of enteral ventilation therapy remains widely debated.^8^ Several groups recently revisited the potential of the enteral route for the systemic delivery of oxygen. Using a liquid solvent called perfluorocarbons with extraordinary oxygen solubility, Okabe et al. showed that intrarectal administration of oxygenated perfluorocarbons led to systemic oxygenation in animals with respiratory failure.^9^ Another preprint study by Mountford et al. demonstrated that delivering oxygen microbubbles increased systemic blood oxygen levels in smoke-inhaled hypoxic pig models.^10^ While the methods above seem promising, GI-mediated systemic oxygenation is compounded by the recipient’s remaining lung function. Therefore, it is imperative to understand parameters that influence the gas exchange efficiency and exclude lung contribution to determine the gut-mediated oxygenation potential.

Herein, we evaluated the effectiveness of the enteral ventilation approach under controlled mechanical ventilation. To dissociate contributions from native lung function, we applied a muscle relaxant in a stable hypoxic minipig model. We investigated whether GI-mediated systemic oxygenation is delivered exclusively from the gut or whether the impact is combined with improved native lung function. Arterial pressure, pulmonary arterial pressure, oxygen saturation (SpO_2_), heart rate, blood temperature, and mixed venous oxygen saturation (SvO_2_) were continuously monitored. The objective of this study was to evaluate the extrapulmonary ventilation pathway in a large animal model to set the stage for future clinical development.

## Results

**Figure 1:**
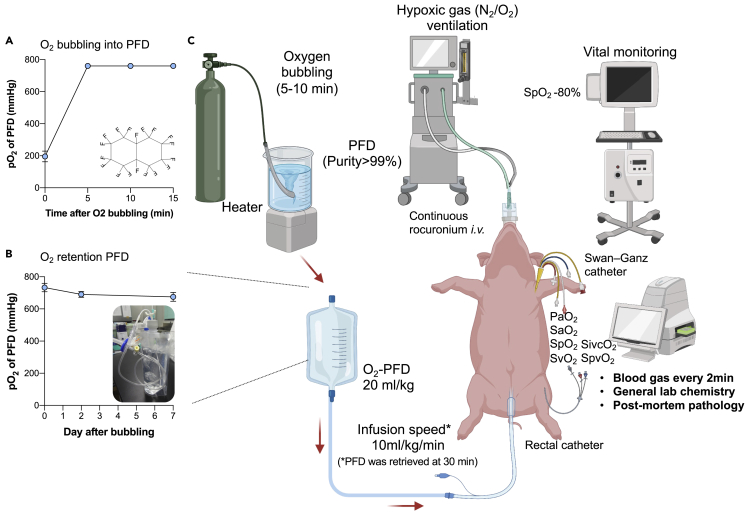
Overview of the experimental protocol using enteral ventilation (A) Oxygen loading capacity of PFD. (B) Oxygen retention capability of the oxygen-loaded PFD in an administration bag. (C) Schematic diagram of the experimental protocols. Rectal O_2_-PFD administration was repeated 3 times, accompanied by collection twice. Data are represented as mean ± SD N_2_, nitrogen; O_2_, oxygen; O_2_-PFD, oxygenated perfluorodecalin; min, minutes; PO_2_, partial pressure of oxygen; SpO_2_, pulse oximetry; PaO_2_, partial pressure of oxygen in arterial blood; SaO_2_, arterial oxygen saturation; SvO_2_, mixed venous oxygen saturation; SivcO_2_, oxygen saturation of the inferior vena cava; SpvO_2_, oxygen saturation of the portal vein.

The experimental outlines are illustrated in Figure 1. The profiles of the nine pigs are summarized in Table 1. We used two differently sized pigs in our experiments to evaluate whether body weight affects the oxygenation effect. In addition, we conducted experiments to confirm the local oxygenation using normal-sized pigs due to the practicality of portal vein cannulation. To introduce hypoxia, FiO_2_ was set at 0.11–0.15. At baseline, SaO_2_, PaO_2_, and PaCO_2_ were 82.5±7.3%, 54.5±6.4mmHg, and 38.0±5.6 mmHg, respectively. Other parameters such as body temperature and blood pressure that might affect the oxygenation curve were carefully controlled throughout the procedures. We bubbled pure oxygen gas into the perfluorodecalin (PFD) liquid to load oxygen into PFD. The partial pressure of oxygen in PFD reached near saturation (760 mmHg) 5 min after the bubbling (Figure 1A). Oxygen levels in PFD were retained at high levels (>650 mmHg) for one week in an administration bag (Figure 1B). Intrarectal administration of oxygenated PFD (O_2_-PFD) was repeated three times in each pig, and 27 experiments were performed.

**Table 1:** Baseline characteristics of hypoxic minipigs before O_2_-PFD administration

	female minipigs (n*=7*)	female pigs (n*=2*)
Weight (kg)	24.2 ± 2.0	42.4 ± 0.8
Mean arterial blood pressure (mmHg)	111 ± 18	79 ± 6
Heart rate (beat/min)	123 ± 16	164 ± 4
Blood temperature (°C)	34.4 ± 0·9	34.3 ± 0.1
Cardiac output (L/min)	1.6 ± 0.7	4.3 ± 0.2
Use of vasopressor Epinephrine Phenylephrine	5 (71%) 3 (43%)	2 (100%) 0 (0%)
FiO_2_	0.13 [0.12–0.15]	0.12 [0.11–0.12]
PEEP (mmHg)	10 [10–10]	10 [10–10]
Respiratory rate (breaths/min)	10 [8–10]	10 [10–10]
SpO_2_ (%)	84.9 ± 4.8	83.0 ± 0.0
SaO_2_ (%)	82.9 ± 11.9	81.6 ± 0.5
PaO_2_ (mmHg)	62.7 ± 12.5	50.4 ± 0.6
PaCO_2_ (mmHg)	38.9 ± 4.0	40.1 ± 0.6

Values are presented as mean ± SD, median [IQR], or number (proportion).

Abbreviations: FiO_2_, fraction of inspiratory oxygen; IQR, interquartile range; O_2_-PFD, oxygenated perfluorodecalin; PaCO_2_, partial pressure of carbon dioxide in arterial blood; PaO_2_, partial pressure of oxygen in arterial blood; PEEP, peak end-expiratory pressure; SaO_2_, arterial oxygen saturation; SD, Standard Deviation SpO_2_, pulse oximetry.

### Correlative factors for oxygenation

Throughout the experiments, immediate oxygenation response was detectable after O_2_-PFD administration in minipigs. Therefore, we first evaluated the early oxygenation dynamics, noting that oxygen transfer peaked around the first 4 min. Mean 4 min-increase in SvO_2,_ SaO_2_, and PaO_2,_ from the baseline was +4.56%, +3.65%, and +3.60 mmHg, respectively. We next explored the correlation between time-evolving oxygenation parameters (at 2, 4, 8, and 12 min after the administration) and the pre-treatment gas parameters to understand the variables for defining oxygen transfer efficiency. All the Pearson correlation coefficients are shown in Figure S1A. For example, the baseline PaO_2_ level showed an inverse correlation with a 4 min-increase in arterial oxygenation (delta (Δ)PaO_2_ 4min) with a correlation coefficient of −0.475. When we limit the baseline PaO_2_ level to 45−65 mmHg, its linearity improves to −0.740. Notably, of all the tested parameters, the baseline SvO_2_ was most closely correlated with the transfer of oxygen, with a linear correlation coefficient of −0.728 with ΔSaO_2_ 4min of O_2_-PFD administration (Figure S1B). These results indicate the enteral ventilation effect starts as early as 2–4 min, and its potency is inversely associated with prior oxygenation status, particularly at the level of the vein.

### Peaked impacts on O_2_ uptake and CO_2_ removal

**Figure 2:**
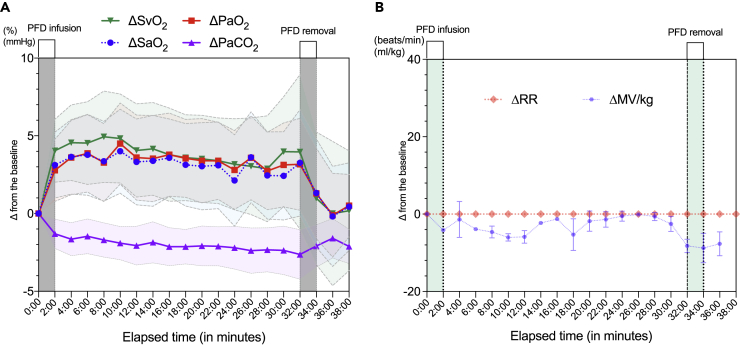
Systemic oxygenation and ventilation effects of enteral ventilation after O_2_-PFD administration and removal (A) Changes in time series of SaO_2_, SvO_2_, PaO_2,_ and PaCO_2_. (B) Time series changes in minute ventilation and respiratory rate. The ventilator was set to mandatory mode with a tidal volume of 8–10 mL/kg and a respiratory rate of approximately 35 mmHg for end-tidal carbon dioxide. The peak end-expiratory pressure (PEEP) was set at 8–10 mmHg. Delta represents the difference in blood gas analysis and respiratory monitoring values before and after O_2_-PFD administration. The red, black, blue, and pink lines represent the mean values of PaO_2_, SaO_2_, SvO_2_, and PaCO_2_, respectively in (A). Each shaded area represents the 95% confidence interval. O_2_-PFD, oxygenated perfluorodecalin; SaO_2_, arterial oxygen saturation; SvO_2_, mixed venous oxygen saturation; PaO_2_, partial pressure of oxygen in arterial blood; PaCO_2_, partial pressure of carbon dioxide in arterial blood; RR, respiratory rate; MV, minute ventilation.

**Figure 3:**
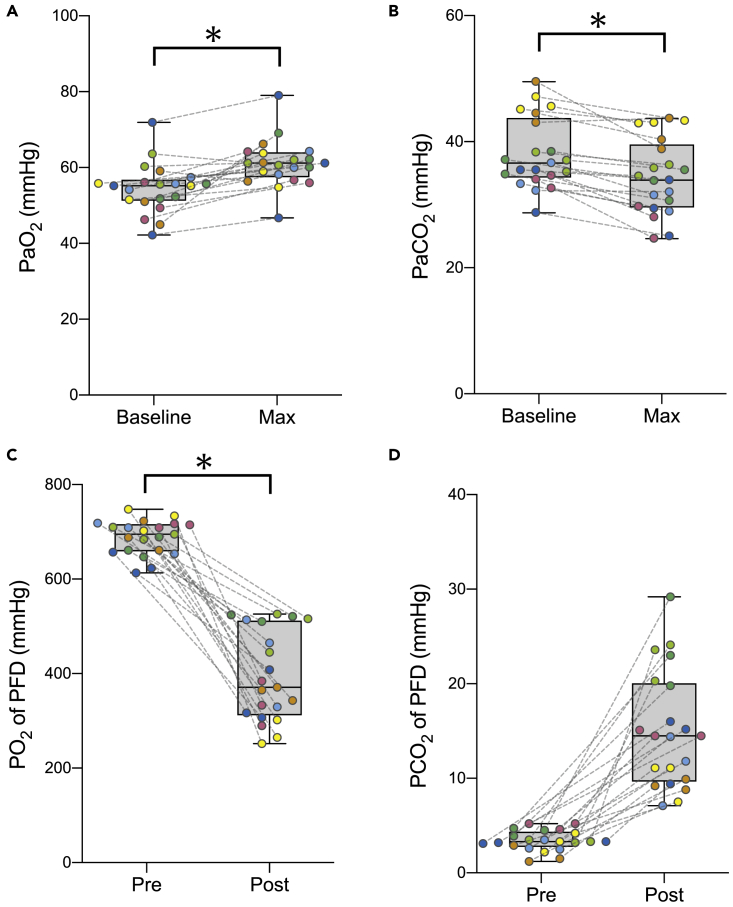
Changes in the partial pressure of oxygen and carbon dioxide before and after enteral ventilation (A) Maximum changes in PaO_2_. (B) Maximum changes in PaCO_2_. (C) Changes in PO_2_ of O_2_-PFD. (D) Changes in PCO_2_ of O_2_-PFD. The box and whisker plots (min to max) are shown. Colored dots represent data from the same pig to show within-/between-animal variations. The p *values* were calculated using a paired t-test. ∗p *< 0*.*001*. O_2_-PFD, oxygenated perfluorodecalin; PaO_2_, partial pressure of oxygen in arterial blood; PaCO_2_, partial pressure of carbon dioxide in arterial blood; PO_2_, partial pressure of oxygen; PCO_2_, partial pressure of carbon dioxide.

We next determined the time-course dynamics of each gas parameter every 2 min in minipigs. The enteral ventilation effect to improve oxygenation was sustained for up to 30 min until PFD was removed from the gut (Figure 2A), while respiratory rate and minute ventilation volume remained unchanged (Figure 2B). The maximum effect on systemic oxygenation was 88.2 ± 5.4% compared to the baseline of 82.5 ± 7.3 (p < 0.001) for SaO_2_. PaO_2_ also increased from 54.5 ± 6.4 to 61.1 ± 6.2 mmHg (p < 0.001), indicating the resolution of the induced respiratory failure conditions (Figure 3A). Given that PFD dissolves significant amounts of most gases, including carbon dioxide, typically in the 120−250 vol % range, *i*.*e*., 3–5 times more than oxygen, we tested if enteral O_2_-PFD has effects on carbon dioxide removal. Interestingly, PaCO_2_ was significantly reduced throughout the experiment: 38.0 ± 5.6 at the baseline and 34.4 ± 5.9 mmHg at post-O_2_-PFD administration (p < 0.001) (Figure 3B). In particular, the increase in oxygenation showed a higher trend in mixed venous blood than in arterial blood throughout treatment (3.4 ± 5.5% in ΔSaO_2_ versus 4.9 ± 6.3% in ΔSvO_2_, at 8 min after O_2_-PFD infusion, p = 0.082), suggesting that the oxygen uptake precedes the arterial oxygenation via venous route.

Such systemic ventilatory effects were rapidly reversed upon removal of the enteral PFD in 30 min. To assess the changes in the dissolved oxygen and carbon dioxide in PFD, we measured the partial pressure of the collected PFD. The oxygen partial pressure was significantly lower after 30 min of administration (from 688 ± 36 to 395 ± 96 mmHg after collection; p < 0.001), whereas the carbon dioxide partial pressure was increased (3.4 ± 1.1 to 15.0 ± 6.2 mmHg after collection; p < 0.001) (Figures 3C and 3D). Conversely, SaO_2_ decreased from 89.0 ± 5.0% to 81.2 ± 7.9% (p < 0.001), and PaO_2_ decreased from 61.1 ± 6.5 to 53.6 ± 7.0 mmHg (p < 0.001), while PaCO_2_ increased from 34.8 ± 5.9 to 37.9 ± 6.7 mmHg (p < 0.001). The post-retrieval trend of rapid oxygen reduction was similar to the change at the time of O_2_-PFD administration (Figure 2A). These results indicate that oxygen and carbon dioxide were exchanged by enteral exposure to PFD.

### Venous oxygenation through the distal intestine

Inspiratory and exhaled oxygen concentrations were stabilized before the administration of O_2_-PFD. Respirator settings remained unchanged throughout the procedures (Figure 2B) and inspiratory oxygen concentration was consistent (FiO_2_, from 12.9 ± 1.7% to 13.0 ± 1.7% (p = 0.164)). However, the exhaled oxygen concentration was increased from 8.1 ± 1.3% (baseline) to 8.9 ± 1.4% (maximum) (p < 0.001) following the administration of O_2_-PFD. The resultant difference between inspiratory and exhaled oxygen concentration reduced from 4.8 ± 0.9% to 4.1 ± 0.9% (p < 0.001). A significant increase in exhaled oxygen concentration indicates that enteral ventilation might oxygenate via returning, *i*.*e*., venous, circulatory route.

**Figure 4:**
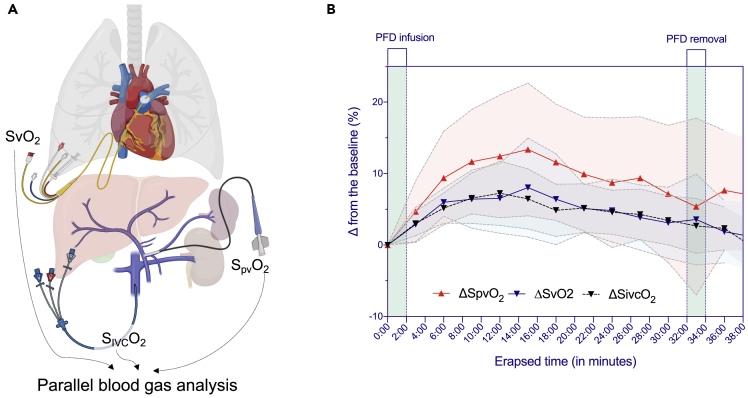
Differences in the effect of oxygenation by enteral ventilation between venous and arterial blood after O_2_-PFD administration (A) A schematic diagram of the venous blood sample at different anatomical levels. (B) Effect of oxygenation in each blood. Delta represents the difference in blood gas analysis values before and after O_2_-PFD administration. The red, black, and blue lines represent the mean values of S_pv_O_2_, S_ivc_O_2_, and SvO_2_, respectively. Each shaded area represents the 95% confidence interval. O_2_-PFD, oxygenated perfluorodecalin; IVC, inferior vena cava; PV, portal vein; S_ivc_O_2_, oxygen saturation of the inferior vena cava; S_pv_O_2_, oxygen saturation of the portal vein; SvO_2_, mixed venous oxygen saturation.

To further explore how enteral ventilation impacts venous oxygenation dynamics in blood at different anatomical levels, we performed the gas analysis of the pulmonary artery, IVC, and portal vein, before and after treatment in normal-sized pigs (Figure 4A). Colorectal venous drainage systems are governed by the superior, middle, and inferior rectal (hemorrhoidal) veins. The superior rectal and inferior mesenteric veins drain into the portal vein, passing the blood through the liver before reaching the systemic circulation. In contrast, the inferior and middle rectal veins drain into the inferior vena cava and, therefore, directly into the systemic circulation, which is approximately responsible for −50% of suppository drug absorption.^11^ Consequently, we determined the contributions of venous oxygenation at the level of the portal vein and the infrarenal level of IVC. At 15 min after O_2_-PFD infusion, S_pv_O_2_ has increased from 55.5 ± 5.9% (baseline) to 68.9 ± 4.3% whereas S_ivc_O_2_ increased from 62.1 ± 2.9% (baseline) to 68.6 ± 5.1%. The oxygenation effect at 15 min ΔS_pv_O_2_ in the portal vein relative to ΔS_ivc_O_2_ was 13.4 ± 7.9% vs. 6.5 ± 3.6% (p = 0.033) (Figure 4B). Interestingly, the venous oxygen saturation in both the portal vein and inferior vena cava showed upward trends until 14 min and then gradually declined while sustained higher level than the baseline (Figure 4B), indicating local oxygen imbalance between supply and consumption occurred in 14–16 min. Thus, enteral ventilation delivers oxygen through the venous system leading to systemic oxygenation in the arterial system.

### Adverse events

The gross examination showed no signs of colorectal damage or perforation after repeated dosing. Consistently, histopathological examination revealed no damage or inflammation to the rectal mucosa due to PFD (Figures S2A–SC). In addition, no macrophages that phagocytized PFD were observed in the spleen (Figures S2D and S2E).

During enteral ventilation under hypoxia, hemodynamics studies showed that mean arterial pressure increased and pulse rate decreased significantly. Maximal vital sign changes induced by O_2_-PFD administration ranged from 115 ± 27 to 129 ± 26 mmHg (p < 0.001) in the mean arterial pressure (MAP) and 117 ± 17 to 112 ± 14 beats/min (p = 0.002) in the pulse rate. After retrieval of the O_2_-PFD from the intestine, the mean blood pressure and pulse rate almost returned to that observed before O_2_-PFD administration (Figure S3). It is not clear whether MAP increase effect is due to an oxygenating effect or an effect of an increased venous return. Thus, despite severe hypoxia, the animals did not undergo shock, and, in fact, the mean arterial pressure increased by an average of approximately 10 mmHg.

## Discussion

Our study, using hypoxic pig models with a combination of approved medical devices and a clinically feasible dose of O_2_-PFD, provides definitive proof that enteral ventilation is possible, independent of the effect of native lung respiration. Initial oxygen transfer and carbon dioxide reduction started within 2 min of O_2_-PFD administration and sustained blood gas status until PFD was removed at 30 min. SvO_2_ dynamic monitoring data indicated that oxygenation most likely started from the venous outflow of the gut, as expected. We also found an increase in oxygen in the portal vein, suggesting potential uptake through the broader large intestine and the inferior mesenteric vein route, even above the level of the rectum. Therefore, extrapulmonary respiration induces systemic oxygenation through venous oxygenation.

Among aquatic organisms that utilize GI as extrapulmonary respiratory organs, the loach has a gas exchange function in its hindgut (distal intestine).^12^^,^^13^ Such gut air-breathing (GAB) species use their gastrointestinal system to maintain systemic oxygenation under respiratory distressed conditions.^14^ Though precise mechanisms for hypoxia acclimation are unclear, aerial ventilation in GAB fishes is driven primarily by oxygen partial pressure of the water (PO_2_).^14^ For example, the onset of aerial respiration occurred at inspired oxygen tensions (P_i_O_2_) between 50 and 60 mmHg, and breathing frequency is linearly increased to 25 mmHg without significant hypometabolic change in *Hypostomus regani*.^15^ Our pig study similarly suggested that the prior hypoxic state influences the mammalian ventilatory effect. Indeed, the oxygen transfer efficiency linearly upregulated in the range of 45–65 mmHg PaO_2_. In support, several reports of intestinal^16^ or peritoneal^17^ perfusion of oxygenated perfluorocarbon, albeit with incremental (-hourly) effectiveness, discussed that the most effective oxygenation was achieved at a pre-treatment PaO_2_ level of around 40–60 mmHg.

Interestingly, the correlation coefficient analysis indicates that the SvO_2_ baseline is inversely associated with systemic oxygenation efficiency relative to pre-treatment arterial oxygenation status. This is in line with our mechanistic expectation since O_2_-PFD is initially exposed to venous circulation via the intestinal mucosa, thereby directly affecting oxygen transfer. Perfluorocarbon-dissolved O_2_ is immediately available to deliver into hemoglobin by diffusion-based mechanisms.^18^ With the intact intestinal epithelial cells, our experimental data favor that diffusion-based oxygen transfer into hemoglobin is a mechanism for the venous oxygen increase. Thus, ventilatory drive in mammalian enteral breathing likely involves recipient oxygenation status and should be considered when developing a clinical application for diseases of hypoxia.

Oxygen carriers for enteral ventilation include oxygen gas, oxygen microbubbles, and perfluorocarbons. Previous studies showed oxygen gas infusion requires mucosal damage to deliver sufficient oxygen^9^ and, therefore, is unacceptable for humans. While oxygenated microbubble infusion is effective when given as a relatively high-dose bolus injection (*i*.*e*., 75–100 mL/kg: 3,750–5,000 mL for 50 kg),^10^ the perfluorocarbon approach can deliver the benefit with modest, repeated, dosing (*i*.*e*., 20 mL/kg: 1000 mL for 50 kg). This injection volume has been within dosage readily approved for human enterography procedures in single-contrast technique. In this study, O_2_-PFD has been slowly infused by drop from a height within 50 cm to prevent a substantial increase in intrarectal pressure. However, the effects of O_2_-PFD on humans are unknown. Therefore, preclinical studies are planned to confirm the tolerability of intrarectal PFD in humans. Of the perfluorocarbon compounds, we elected to use perfluorodecalin (PFD), a perfluorocarbon already extensively used in animals^9^ and human studies involving blood substitutes, liquid breathing, eye surgery,^19^ and known for its extraordinary oxygen and carbon dioxide dissolving capacities. Consistent with the reported safety of PFD in humans, enteral O_2_-PFD exposure is safe and tolerable without significant complications such as colorectal perforation, bacterial translocation, or liver dysfunction.

Intrarectal bolus infusion of O_2_-PFD can be operated without a specialized respiratory care team even in resource-limited settings. Such affordable technique will provide a supplemental way to oxygenate until advanced medical care is available as a bridging therapy. Some potential scenarios include the prehospital transport, emergency, or the preparatory settings for the invasive respiratory support.

### Limitations of the study

This study, however, has a few limitations. First, the observation was confined for up to 30 min; therefore, the duration of efficacy and cumulative risk is unclear. Second, PFD dose-dependent effect was not tested. Third, respirator settings remained constant throughout the procedures, precluding the assessment of the crosstalk between intra- and extrapulmonary respirations effects. Finally, since this study was carried out in pigs under general anesthesia and muscle relaxant, tolerability under an awake status requires future evaluation. Nevertheless, our present study, conducted using a combination of clinically relevant medical devices, including PFD and a rectal balloon tube, firmly supports the therapeutic potential of extrapulmonary ventilation. Further preclinical and clinical development of the enteral ventilation approach is warranted.

## STAR★Methods

### Key resources table

**Table:** 

REAGENT or RESOURCE	SOURCE	IDENTIFIER
**Chemicals, peptides, and recombinant proteins**		

Perfluorodecalin	F2 Chemicals Ltd. Shandong Zhongshan Photoelectric Materials Co., Ltd.	Cas: 306-94-5
Ketamine	Daiichi Sankyo Propharma Co.,Ltd.	Cas: 1867-66-9
Xylazine	Bayer Yakuhin, Ltd.	Cas: 7361-61-7
Isoflurane	Mylan N.V.	Cas: 26675-46-7
Rocuronium	MSD	Cas: 119302-91-9
Adrenaline	Daiichi Sankyo Co,Ltd.	Cas: 51-43-4
Phenylephrine	Kowa Co., Ltd.	Cas: 61-76-7
Lactated Ringer’s Solution	Otsuka Pharmaceutical Co., Ltd.	CID: 56841910
Niflec® Combination Powder	EA Pharma Co., Ltd.	N/A

**Experimental models: Organisms/strains**		

minipig	IVTeC Co. Ltd./Fuji Micra, Inc.	N/A
Pig	IVTeC Co. Ltd./Fuji Micra, Inc.	N/A

**Software and algorithms**		

GraphPad Prism 9	Graphpad Software	https://www.graphpad.com/
R version 4.1.2	R Project	https://www.r-project.org

**Other**		

anesthesia machine	ACOMA Medical Industry	FO-20A
ventilator	ACOMA Medical Industry	Spiritus
double-balloon rectal catheter	Create Medic CO., LTD.	N/A
bedside patient monitor	Nihon Kohden Corporation	Life Scope VS, BSM-3592
multigas analyzer	Nihon Kohden Corporation	GF-119P
blood gas analyzer	Radiometer Medical ApS	ABL90 FLEX
pulmonary artery catheter	Edwards Lifesciences Corporation	631F55N
hemodynamic monitor	Edwards Lifesciences Corporation	Vigilance II

### Resource availability

#### Lead contact

Further information and requests for resources and reagents should be directed to, and will be fulfilled by the lead contact, Takanori Takebe (takanori.takebe@cchmc.org)

#### Materials availability

This study did not generate any new materials or reagents.

### Experimental model and subject details

#### Animals

Experiments in pigs were carried out in the laboratory of IVTeC Co., Ltd. (Kobe, Japan) and were approved by the Association for the Assessment and Accreditation of Laboratory Animal Care International. The experimental protocols (#IVT21-179/IVT22-10) were approved by IVTeC Co. Ltd. Female minipigs (21.2–26.4 kg, n = 7) and female normal-sized pigs (41.6–43.2 kg, n = 2) were fasted for two days and given free access to water. A peroral intestinal irrigator (Niflec®, EA Pharma Co., Ltd., Tokyo, Japan) was administered as a laxative two days before the experiment.

#### Hypoxia model

The hypoxic pig model was established by mixing oxygen and nitrogen using a gas flow meter in an anesthesia machine (FO-20A, ACOMA Medical Industry Co., Ltd., Tokyo, Japan). The inspiratory oxygen fraction (FiO_2_) was adjusted to maintain the pulse oximeter (measuring SpO_2_) at approximately 85%. Arterial blood gas analysis was initiated 10 min before experimental manipulation to confirm arterial oxygen saturation (SaO_2_), partial pressure of oxygen in arterial blood (PaO_2_), and partial pressure of carbon dioxide in arterial blood (PaCO_2_) were stable.

### Method details

#### Anesthesia, maintenance, and monitoring

A mixture of ketamine (10 mg/kg, intramuscular), xylazine (2 mg/kg, intramuscular), and isoflurane were used as anesthetics. After tracheal intubation, 2% isoflurane was used to maintain general anesthesia. After administering an intravenous bolus of rocuronium (25 mg), controlled mechanical ventilation was initiated under continuous intravenous rocuronium infusion (100 mg/h) (MSD Co. Ltd, Tokyo, Japan). To maintain the intravenous fluids, Ringer’s lactate solution was administered at a dose of 5 mL/kg/h. In the case of pre-experimental hemodynamic instability, a 100 mL Ringer’s lactate solution bolus was administered intravenously, and the maintenance fluid dose was increased to 10 mL/kg/h. If hemodynamic instability persisted after fluid loading, phenylephrine and/or epinephrine were continuously administered. Enteral ventilation protocol was initiated after the hemodynamics stabilized for more than 10 minutes.

The hypoxic pigs did not breathe spontaneously under continuous administration of high-dose muscle relaxants and were mechanically ventilated under controlled conditions. The ventilator (Spiritus; ACOMA Medical Industry Co., Ltd., Tokyo, Japan) was set at a tidal volume of 8–10 mL/kg and a respiratory rate of approximately 35 mmHg for end-tidal carbon dioxide. The peak end-expiratory pressure (PEEP) was set at 8–10 mmHg in the 5° head-up position to prevent deterioration of the lung condition, e.g., due to atelectasis. The ventilator settings remained constant throughout all experimental processes.

Catheters were placed in the right carotid and femoral arteries for repeated blood sampling and blood pressure monitoring, respectively. A Swan–Ganz catheter was placed in the pulmonary artery through the right jugular vein under fluoroscopy. In separate experiments, a central venous catheter was placed at the infrarenal level of the posterior vena cava (inferior vena cava [IVC]) under fluoroscopy, and a portal vein catheter was placed under direct guidance via laparotomy.

Arterial pressure, pulmonary arterial pressure, SpO_2_, heart rate, blood temperature, and mixed venous oxygen saturation (SvO_2_) were continuously monitored using a bedside patient monitor (Life Scope VS BSM-3592, Nihon Kohden Corporation, Tokyo, Japan). Cardiac output was measured intermittently using bolus thermodilution methods through a Swan-Ganz catheter before and after the intervention.

#### Enteral ventilation and blood gas analysis

Perfluorodecalin (PFD) (F2 Chemicals Ltd, Lea Town, Lancashire, UK or Shandong Zhongshan Photoelectric Materials Co., Ltd, Shandong, China) was used as the perfluorocarbon. Oxygenated perfluorodecalin (O_2_-PFD) was prepared by 3 L/min pure oxygen bubbling for at least 10 min. The oxygen partial pressure of O_2_-PFD was confirmed to be >600 mmHg before the experiment. Subsequently, a rectal balloon tube (Create Medic Co., Ltd., Yokohama, Japan) was placed in the porcine rectum, and the catheter balloon was inflated and fixed. O_2_-PFD (20 ml/kg) was administered into the distal intestine by a rectal tube for approximately 2 min. Arterial and pulmonary arterial blood gas analyses were performed simultaneously every 2 min from the start of O_2_-PFD infusion for 30 min. O_2_-PFD was retrieved from the intestine through a rectal tube 30 min after O_2_-PFD infusion. Arterial blood gas analyses were performed every 2 min for 10 min after O_2_-PFD collection. This procedure was repeated three times. The experimental protocol is illustrated in Figure 1.

In an additional experiment, O_2_-PFD (20 ml/kg) was administered into the distal intestine through a rectal balloon catheter (Create Medic Co., Ltd., Yokohama, Japan). Arterial, IVC, and portal vein blood samples were analyzed simultaneously every 3 minutes for up to 30 minutes after O_2_-PFD infusion. O_2_-PFD was collected by spontaneous discharge from the anus. Arterial blood gas analyses were performed every 3 min until 15 min after O_2_-PFD collection. These procedures were performed twice on each pig.

All blood gas analyses were performed using an ABL90 FLEX blood gas analyzer (Radiometer Medical ApS, Copenhagen, Denmark) equipped with a co-oximeter.

#### Outcome measurements

The effect of systemic oxygenation and ventilation were measured using arterial and pulmonary arterial blood gas analyses. Increases in oxygen saturation (SaO_2_, SvO_2_) and arterial partial pressure (PaO_2_, PaCO_2_) from baseline before administration of O_2_-PFD were evaluated. For each blood oxygenation parameter (PaO_2_, SaO_2_, SvO_2_), the correlation between baseline status and oxygenation was assessed at each measurement interval after O_2_-PFD administration. The oxygen and carbon dioxide partial pressures of PFD were also measured before and after intestinal administration of O_2_-PFD.

To assess venous oxygenation levels, the portal vein and infrarenal level of IVC blood samples and pulmonary arterial blood samples were analyzed in an additional experiment using normal-sized pigs. Inspiratory oxygen and expiratory oxygen concentrations were measured by monitoring anesthetic and respiratory gases to evaluate oxygen discharge from expiration rather than inspiration (GF-119 Multi-Gas Unit, Nihon Kohden Corporation, Tokyo, Japan).

We used postmortem histopathology to assess whether the intestinal mucosa and spleen were damaged as a side effect of O_2_-PFD infusion. Vital signs, such as mean arterial pressure and pulse rate, were also monitored during the procedures.

### Quantification and statistical analysis

The baseline characteristics of hypoxic pigs were compared using Student’s *t* test, Mann–Whitney U test, or Fisher exact test. Systemic oxygenation and maximum ventilation effect by blood gas analysis before and after O_2_-PFD administration was evaluated using a paired *t*-test. The simultaneous increase in the oxygenation rate in arterial and mixed venous blood from baseline was compared using the Wilcoxon signed-rank test. The Pearson correlation coefficient evaluated the relationship between the pre-treatment condition and blood gas parameters after O_2_-PFD administration. The difference between inspiratory and exhaled oxygen concentrations was analyzed using a paired t-test. Categorical variables were expressed as numeric values (proportion), and continuous variables were presented as mean ± standard deviation (SD) or median (interquartile range [IQR]). Delta (Δ) represents the difference in blood gas analysis values before and after O_2_-PFD administration. Statistical significance was set at p < 0.05. All statistical analyses were performed using Graph Pad Prism9 software and R software, version 4.1.2 (The R Foundation for Statistical Computing, Vienna, Austria).

## Data Availability

• Data reported in this paper will be shared by the lead contact upon request. • This study did not generate new codes. • Any additional information required to reanalyze the data reported in this paper is available from the lead contact upon request.
