# Supplementary material for: Enteral liquid ventilation oxygenates a hypoxic pig model
Source: iScience. 2023 Feb 13;26(3):106142. doi: 10.1016/j.isci.2023.106142 (PMC9984951; doi:10.1016/j.isci.2023.106142)
Supplement: Document S1. Figures S1–S3 [file mmc1.pdf]

iScience, Volume *26*

Supplemental information

Enteral liquid ventilation oxygenates a hypoxic pig model

Tasuku Fujii, Yosuke Yoneyama, Akiko Kinebuchi, Naoki Ozeki, Sho Maeda, Norikazu Saiki, Toyofumi Fengshi Chen-Yoshikawa, Hiroshi Date, Kimitoshi Nishiwaki, and Takanori Takebe

SUPPLEMENTALY INFORMATION

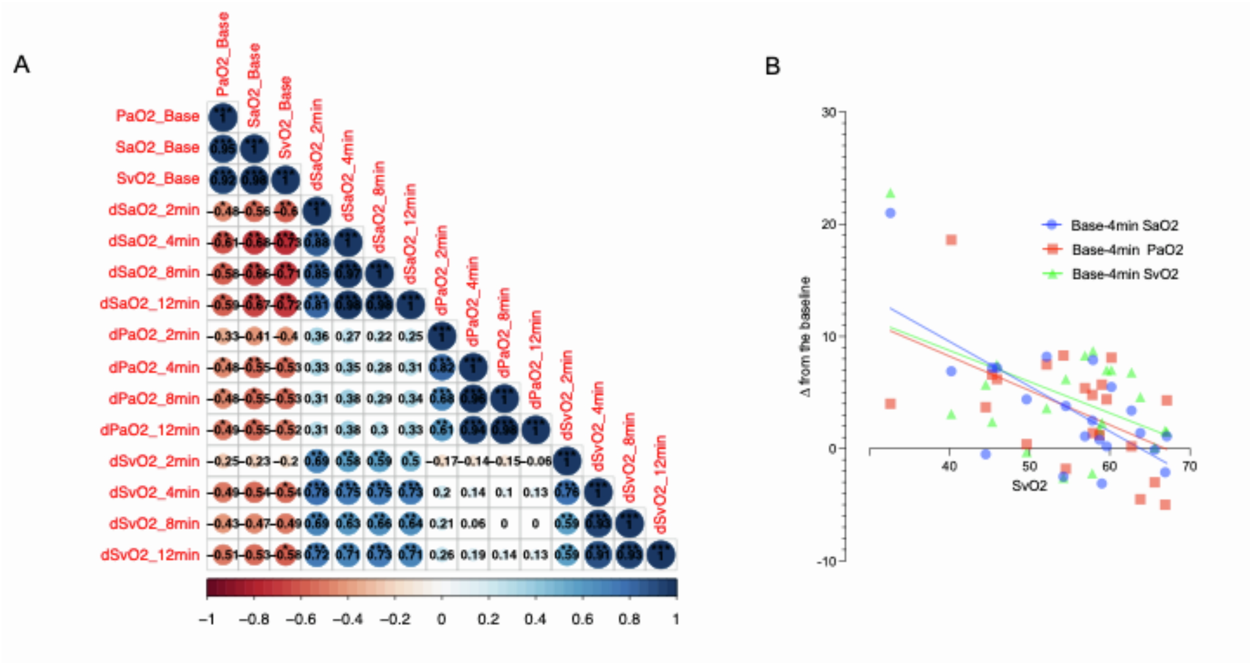

Figure S1. Correlation between the baseline status and oxygenation of each blood oxygenation parameter after O_2-PFD administration, Related to Figure 2.

(A) Correlation matrix of baseline and each blood oxygenation parameter. **(B)** SvO_2 baseline and each blood oxygenation parameter in the first 4 min. Delta or "d" represents the difference in values before and after O_2-PFD administration. SaO_2, arterial oxygen saturation; SvO_2, mixed venous oxygen saturation; PaO_2, partial pressure of oxygen in arterial blood.

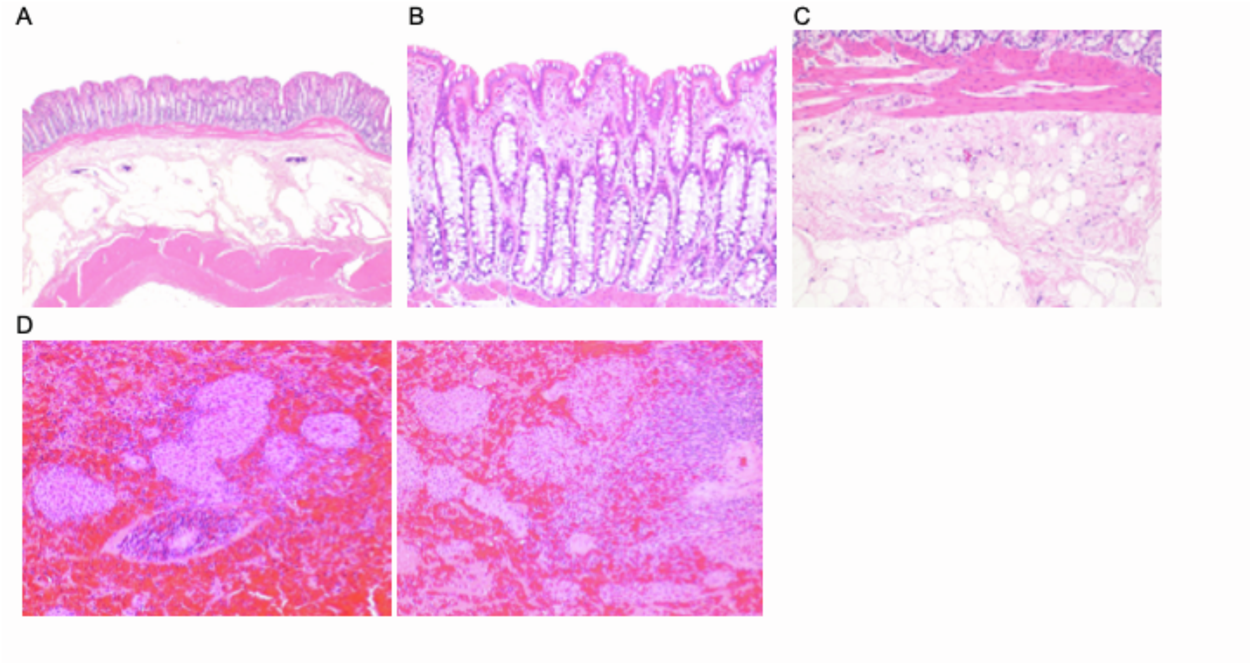

Figure S2. Histopathological examination of the rectal mucosa and spleen after administration of O_2-PFD, Related to Figure 2.

(A) H&E staining of the rectal tissues. (×6.25)

(B) Enlarged image of the rectal mucosal layer. (×50)

(C) Enlarged image of the rectal muscularis mucosa and submucosa. (×50)

(D) H&E staining of the spleen. (×50)

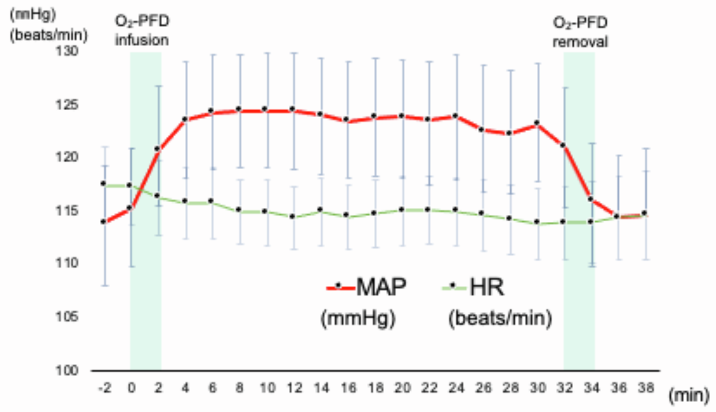

Figure S3. Vital signs of minipigs during enteral ventilation, Related to Figure 2.

The red and black lines indicate the MAP and HR, respectively. Data are represented as mean ± SD. O_2-PFD, oxygenated perfluorodecalin; MAP, mean arterial pressure (mmHg); HR, heart rate, or pulse rate (beats/min) ; SD, standard deviation.
